# Exogenous sphingomyelinase increases collagen and sulphated glycosaminoglycan production by primary articular chondrocytes: an *in vitro *study

**DOI:** 10.1186/ar1961

**Published:** 2006-05-12

**Authors:** Sophie J Gilbert, Emma J Blain, Pamela Jones, Victor C Duance, Deborah J Mason

**Affiliations:** 1 Connective Tissue Biology Laboratories, School of Biosciences, Cardiff University, Museum Avenue, Cardiff, Wales, UK

## Abstract

We previously established a role for the second messenger ceramide in protein kinase R (PKR)-mediated articular cartilage degradation. Ceramide is known to play a dual role in collagen gene regulation, with the effect of ceramide on collagen promoter activity being dependent on its concentration. Treatment of cells with low doses of sphingomyelinase produces small increases in endogenous ceramide. We investigated whether ceramide influences articular chondrocyte matrix homeostasis and, if so, the role of PKR in this process. Bovine articular chondrocytes were stimulated for 7 days with sphingomyelinase to increase endogenous levels of ceramide. To inhibit PKR, 2-aminopurine was added to duplicate cultures. *De novo *sulphated glycosaminoglycan and collagen synthesis were measured by adding [^35^S]-sulphate and [^3^H]-proline to the media, respectively. Chondrocyte phenotype was investigated using RT-PCR and Western blot analysis. Over 7 days, sphingomyelinase increased the release of newly synthesized sulphated glycosaminoglycan and collagen into the media, whereas inhibition of PKR in sphingomyelinase-treated cells reduced the level of newly synthesized sulphated glycosaminoglycan and collagen. Sphingomyelinase treated chondrocytes expressed *col2a1 *mRNA, which is indicative of a normal chondrocyte phenotype; however, a significant reduction in type II collagen protein was detected. Therefore, small increments in endogenous ceramide in chondrocytes appear to push the homeostatic balance toward extracellular matrix synthesis but at the expense of the chondrocytic phenotype, which was, in part, mediated by PKR.

## Introduction

The signalling molecule ceramide belongs to a family of highly hydrophobic molecules containing a variable length fatty acid linked to sphingosine [1]. As well as its established role in membrane structure, many studies have now shown that ceramide is a key second messenger, activating a number of intracellular signalling cascades that are implicated in a wide range of cellular functions such as proliferation, differentiation, necrosis and apoptosis [2-4]. Interestingly, Sabatini and coworkers [5,6] recently implicated ceramide signalling in the regulation of proteoglycan degradation and mRNA expression of matrix metalloproteinases (MMPs) 1, 3 and 13 in rabbit articular chondrocytes. Furthermore, we demonstrated that application of exogenous ceramide induces articular cartilage degradation, which is, in part, mediated through protein kinase R (PKR) [7,8]. Treatment of cartilage explants with the short chain, cell permeable ceramide analogue C_2_-ceramide resulted in PKR-mediated increases in chondrocyte death and release of proteoglycans and pro- and active MMP-2. In addition, ceramide has been shown to activate PKR in leukaemia cell lines, and at high concentrations it results in PKR-mediated inhibition of protein synthesis [4]. Thus, ceramide signalling, via the PKR pathway, may play a pivotal role in articular cartilage metabolism.

**Figure 1:**
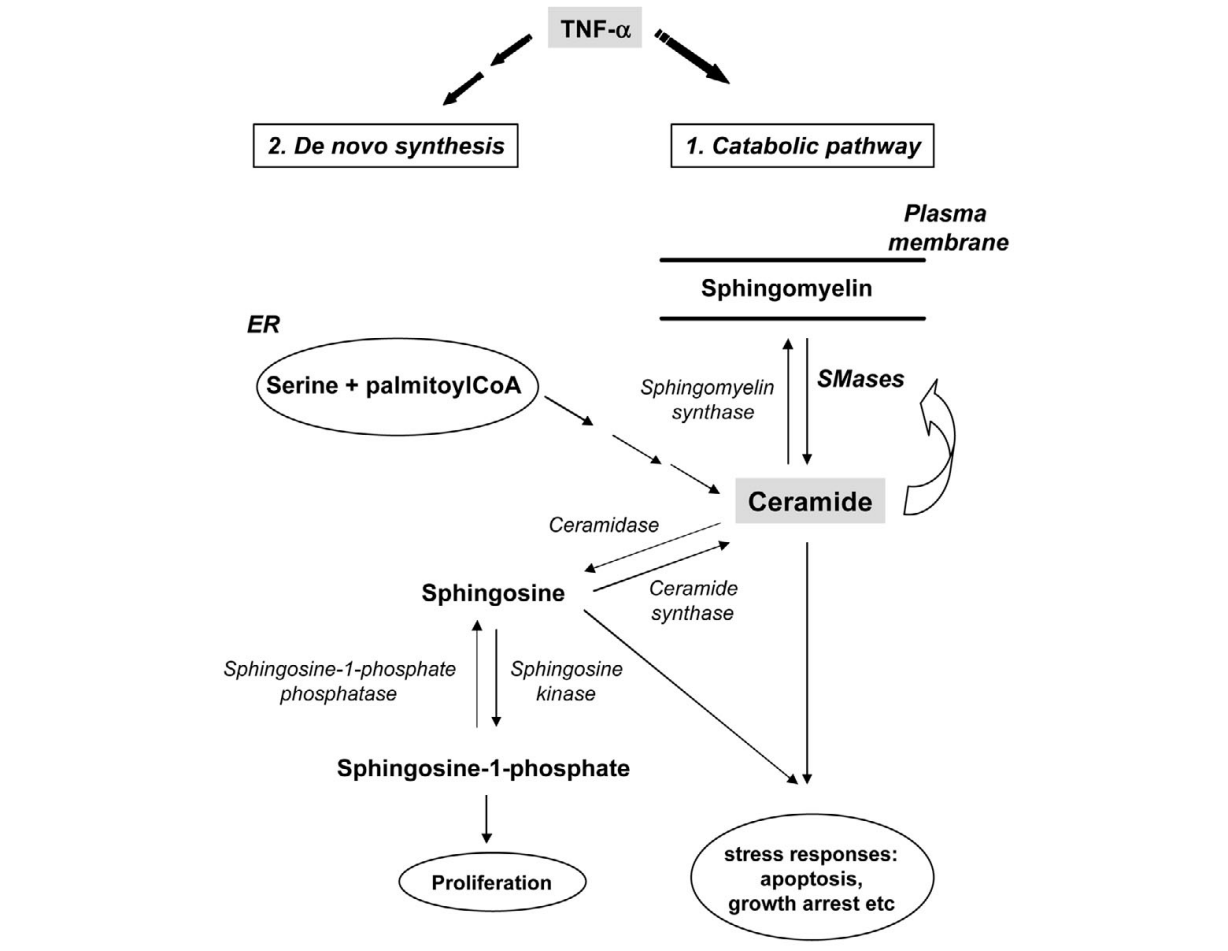
Metabolic pathways involved in the production of endogenous ceramide. Endogenous ceramide is produced via 2 main mechanisms: a catabolic pathway, involving the hydrolysis of the membrane lipid sphingomyelin by endosomal acidic and membrane-bound neutral SMases; and *de novo *synthesis. TNF-α can increase cellular ceramide via both mechanisms. The rise in ceramide can create a positive feedback loop to amplify ceramide production further via the activation of SMases. Once generated, ceramide can transiently accumulate within the cell or be converted into various metabolites such as sphingosine and sphingosine-1-phosphate. Cell responses to ceramide will therefore depend on the engagement of downstream effectors, the cell microenvironment and concomitant activation of enzymes that convert ceramide into other metabolites. CoA, coenzyme A; ER, endoplasmic reticulum; SMase, sphingomyelinase; TNF, tumour necrosis factor.

Endogenous ceramide is produced via two main pathways: the catabolic pathway involving hydrolysis of the membrane lipid sphingomyelin by endosomal acidic and membrane-bound neutral sphingomyelinases (SMases); and *de novo *synthesis [3] (Figure 1). Hydrolysis of sphingomyelin at the external leaflet of the plasma membrane by the application of exogenous bacterial SMase, an enzyme with properties similar to those of neutral SMase, leads to a transient increase in intracellular ceramide formation [9], the magnitude of which increases with increasing doses of SMase [10]. Treatment of cells with tumour necrosis factor (TNF)-α also increases cellular ceramide but in a more sustained manner [10]. Increased levels of intracellular ceramide can create a positive feedback loop to amplify ceramide production further via the activation of endogenous SMases [11]. Once generated, ceramide transiently accumulates within the cell or is converted into various metabolites such as sphingosine and sphingosine-1-phosphate (Figure 1) [12]. Cell responses to ceramide depend upon the engagement of downstream effectors, the cell microenvironment and concomitant activation of enzymes that convert ceramide into other metabolites. In some cell types, raising the intracellular levels of ceramide is sufficient to induce stress responses such as apoptosis and cell cycle arrest [9]. Therefore, within the cell a dynamic balance must exist between the levels of ceramide and sphingosine, which promote antigrowth effects, and sphingosine-1-phosphate, which promotes proliferation (Figure 1) [3,9,12-15]. Ceramidase converts ceramide to sphingosine and thus contributes to this balance [13]. Absence of ceramidase causes Farber's disease, in which an accumulation of excess ceramide within the cartilage and bone leads to joint pain and arthritis-like joint degeneration [16].

Evidence suggests that there is a dual role for sphingolipids in collagen gene regulation, supporting the existence of a sphingolipid rheostat [15]. Low concentrations of ceramide stimulate type I collagen promoter activity in fibroblasts, whereas high concentrations of ceramide potently inhibit collagen gene transcription and decrease collagen protein production in fibroblasts and hepatic stellate cells [17-19]. To our knowledge, no studies have been conducted to investigate the effect of ceramide accumulation on chondrocyte extracellular matrix (ECM) homeostasis. However, the research described above suggests that increases in endogenous ceramide may affect cartilage ECM protein transcription and translation, as well as activating degradative pathways that are involved in the pathogenesis of diseases such as osteoarthritis. The aims of the present study were therefore to investigate the effect of increasing the levels of endogenous ceramide on articular chondrocyte homeostasis and to determine whether any ceramide-induced changes in matrix metabolism are mediated via the PKR signalling pathway.

## Materials and methods

### Materials

All chemicals were obtained from Sigma (Poole, UK) unless otherwise stated and were of analytical grade or above. Culture medium consisted of Dulbecco's modified eagle's medium (DMEM; DMEM-Glutamax-I™, Invitrogen, Paisley, UK) supplemented with 100 U/ml penicillin, 100 μg/ml streptomycin, 50 μg/ml ascorbate-2-phosphate and 1× insulin-transferrin-sodium selenite (ITS). For radiolabelling experiments, DMEM-Glutamax-I™ was replaced with a 1:1 mixture of DMEM-Glutamax-I™ and Hams F12 media.

### Primary articular chondrocyte culture

Articular cartilage was taken from the metacarpalphalangeal joint of 7-day-old calves within 12 hours of slaughter using a scalpel, and full-depth cartilage explants (20–70 mg) were cultured overnight at 37°C in a humidified atmosphere of 5% carbon dioxide and 95% air in 1 ml of DMEM-Glutamax-I™ supplemented with 10% foetal calf serum. DMEM-Glutamax-I™ containing foetal calf serum was removed and chondrocytes isolated as previously described [20]. Following isolation, chondrocytes were cultured (1 × 10^6 ^cells/well of a 24-well plate) overnight at 37°C in serum-free DMEM-Glutamax-I™ supplemented with ITS in order to maintain their chondrocytic phenotype [21] and prevent serum withdrawal activation of signalling pathways [22]. To increase endogenous levels of ceramide, chondrocytes were stimulated for up to 10 days with bacterial SMase (0.1–1.0 U/ml) [10]. Media and treatments were refreshed at 7 days if cultures were extended to 10 days. To investigate the role of PKR in SMase-mediated responses, the PKR inhibitor 2-aminopurine (2AP; 1 mmol/l) was added to duplicate cultures 1 hour before and during the addition of treatments. This concentration inhibits activation of PKR in a number of cell types [4,8,23-26] and does not affect chondrocyte viability [8]. Following treatment, media was removed and stored at -20°C and 200 μl ice-cold extract buffer (0.9% Triton X-100) containing protease inhibitors (1 μmol/l leupepstatin hemisulphate, 150 nmol/l aprotinin, 0.5 mmol/l EDTA disodium salt, 500 μmol/l AEBSF HCl, 1 μmol/l E64; Merck Biosciences, Nottingham, UK) and phosphatase inhibitors (phosphatase inhibitor cocktail set II, according to manufacturer's instructions; Merck Biosciences, Nottingham, UK) was added to the cells. Cell extracts were stored at -80°C for future analysis.

### Cytotoxicity assay and total cell number

Cell death was assessed using the CytoTox 96^® ^assay (Promega, Southampton, UK), which quantitatively measures lactate dehydrogenase (LDH) present in the culture media that has been released upon natural lysis of cells during the culture period [8,27]. This assay measures both primary and secondary necrotic cell death. Differences in the release of LDH associated with culture treatment were expressed as absorbance units. The total cell number, at the end of each treatment, was also determined using the CytoTox 96^® ^assay. This assay can be used to measure indirectly the LDH activity present in the cytoplasm of cells that are intact at the end of the culture period. Cell quantification, therefore, occurs following lysis of the cells by the addition of extract buffer. The number of cells present is directly proportional to the absorbance value, which represents LDH activity [10].

### Analysis of proteoglycan release

The amount of sulphated glycosaminoglycan (sGAG) released into the medium of chondrocyte cultures was measured using the dimethylmethylene blue (DMMB) assay using chondroitin-4-sulphate-C from shark cartilage as a standard, as described previously [28]. Differences in the release of sGAG associated with culture treatment were expressed as micrograms of GAG released per cell.

### Determination of protein concentration

The protein concentration of cell extracts after 24 hours of treatments was determined using the BCA method, in accordance with the manufacturer's instructions (Perbio Science, Cramlington, UK).

### Analysis of *de novo *matrix synthesis using [^35^S]-sulphate and [^3^H]-proline radiolabelling

To measure newly synthesized protein and sGAGs, chondrocytes (4 × 10^5 ^cells/well of a 48-well plate) were treated with sphingomyelinase (0.1 U/ml) in the presence of 20 μCi/ml of [^3^H]-proline and 10 μCi/ml [^35^S]-sulphate (GE Healthcare, Chalfont St Giles, UK). At the end of the treatment period, unincorporated radiolabel was removed from the media and cell extracts using Ultrafree^®^-MC centrifugal filter units, in accordance with the manufacturer's instructions (Millipore, Watford, UK). Incorporated [^35^S] radioactivity in the culture media and cell extracts was counted (Beckman Scintillation Counter; Beckman Coulter, High Wycombe, UK) as a measure of *de novo *sGAG synthesis.

*De novo *collagen synthesis was determined by digesting labelled protein in media and cell extracts with 8U bacterial collagenase (Worthington's Type 3 collagenase; Lorne Laboratories, Reading, UK) overnight at 37°C [29]. Digested collagen fragments were removed using Ultrafree^®^-MC filter units and the remaining undigested [^3^H] counts taken as a measure of noncollagenous protein. Collagenous protein was calculated using the following equation: collagen (counts/min) = total protein (counts before digestion) – noncollagenous protein (counts after digestion).

### RNA extraction, cDNA synthesis and PCR

To investigate chondrocyte phenotype and to determine whether acidic and neutral SMase are expressed in articular chondrocytes, RT-PCR was performed. Chondrocytes were treated with or without SMase (0.1 U/ml), placed into TRIZOL^® ^(1 × 10^6 ^cells/ml) and total RNA was extracted, in accordance with the manufacturer's instructions (Invitrogen, Paisley, UK). RNA samples were DNase (Ambion, Huntingdon, UK) treated to remove genomic DNA, in accordance with the manufacturer's protocol, and resuspended in 50 μl sterile water. cDNA was generated in a single 20 μl reaction from 11 μl RNA sample using 250 ng random hexamers (0.5 mg/ml; Promega) and Superscript II reverse transcriptase (200 units), in accordance with the manufacturer's instructions (Invitrogen). cDNA integrity and lack of genomic DNA contamination were confirmed by PCR using primers to glyceraldehyde-3-phosphate dehydrogenase (GAPDH [GenBank:U85042]; Table 1). PCR primers (Table 1) designed to acidic SMase [GenBank:AF325550], neutral SMase [GenBank:NM031360], type IIA and IIB collagen [30], and Sox9 [GenBank:AF278703] sequences were used to amplify cDNA derived from bovine articular chondrocytes. cDNA or water controls (1 μl) were amplified for 25–30 cycles in a 12.5 μl reaction volume (0.2 units Taq polymerase [Promega], 200 μmol/l of each dNTP, 1.5–2.5 mmol/l MgCl_2 _and 0.2–0.4 μmol/l of each primer; Table 1) using the following cycling parameters: 94°C for 30 s; 58°C or 60°C for 30 s; and 72°C for 1 min. Amplified products were separated alongside a 100 base pair DNA ladder (Promega) on 1–2% agarose gels, containing ethidium bromide (10 μg/ml).

### Quantitative PCR

Type II collagen and aggrecan gene expression were measured by quantitative PCR (qPCR). cDNA was produced as detailed above and qPCR carried out using an ABI 7700 Sequence Detection System, in accordance with the manufacturer's instructions (Applied Biosystems, Warrington, UK) using 300 nmol/l forward and reverse primers and 200 nmol/l probe (5' 6-carboxyfluorescein and 3' 6-carboxytetramethylrhodamine). The GAPDH gene was used as an endogenous control to normalize for differences in the amount of total RNA present in each sample; GAPDH primers (forward: 5'-GGCATCGTGGAGGGACTTATGA-3'; reverse: 5'-CAGAAGACTGTGGATGGCCC-3') and probe (5'-CACTGTCCACGCCATCACTGC-3') were purchased from Applied Biosystems. Primers and probes to type II collagen and aggrecan were as previously described [31].

### Western blot analysis of type II collagen

To further investigate the phenotype of bovine chondrocytes following culture in the presence and absence of SMase, Western blotting was performed, as described previously [32]. Cell associated material and media samples (from equivalent cell numbers) were reduced (5% β-mercaptoethanol) and resolved on 7.5% (weight/vol) SDS-polyacrylamide gels and transferred subsequently to PVDF membrane (Immobilon; Millipore). Binding of our monclonal antibody to type II collagen (AVT6E3) [33] and horseradish peroxidase conjugated anti-mouse IgG was detected using enhanced chemiluminescence reagents (GE Healthcare) on Hyperfilm-ECL (GE Healthcare).

### Statistical analysis

Data are representative of at least three independent experiments except for the radiation experiment, which was repeated twice. Data are presented, following normalization to cell number, as mean ± standard error (*n *≥ 3), tested for normality and equal variances, and analyzed by Student's two-sample *t *test (Minitab Statistical Software; Minitab Ltd, Coventry, UK). Treatments were compared with untreated control cells and differences were considered significant at the 5% level (*P *< 0.05).

## Results

### Effect of increasing doses of exogenous sphingomyelinase on chondrocyte function

**Figure 2:**
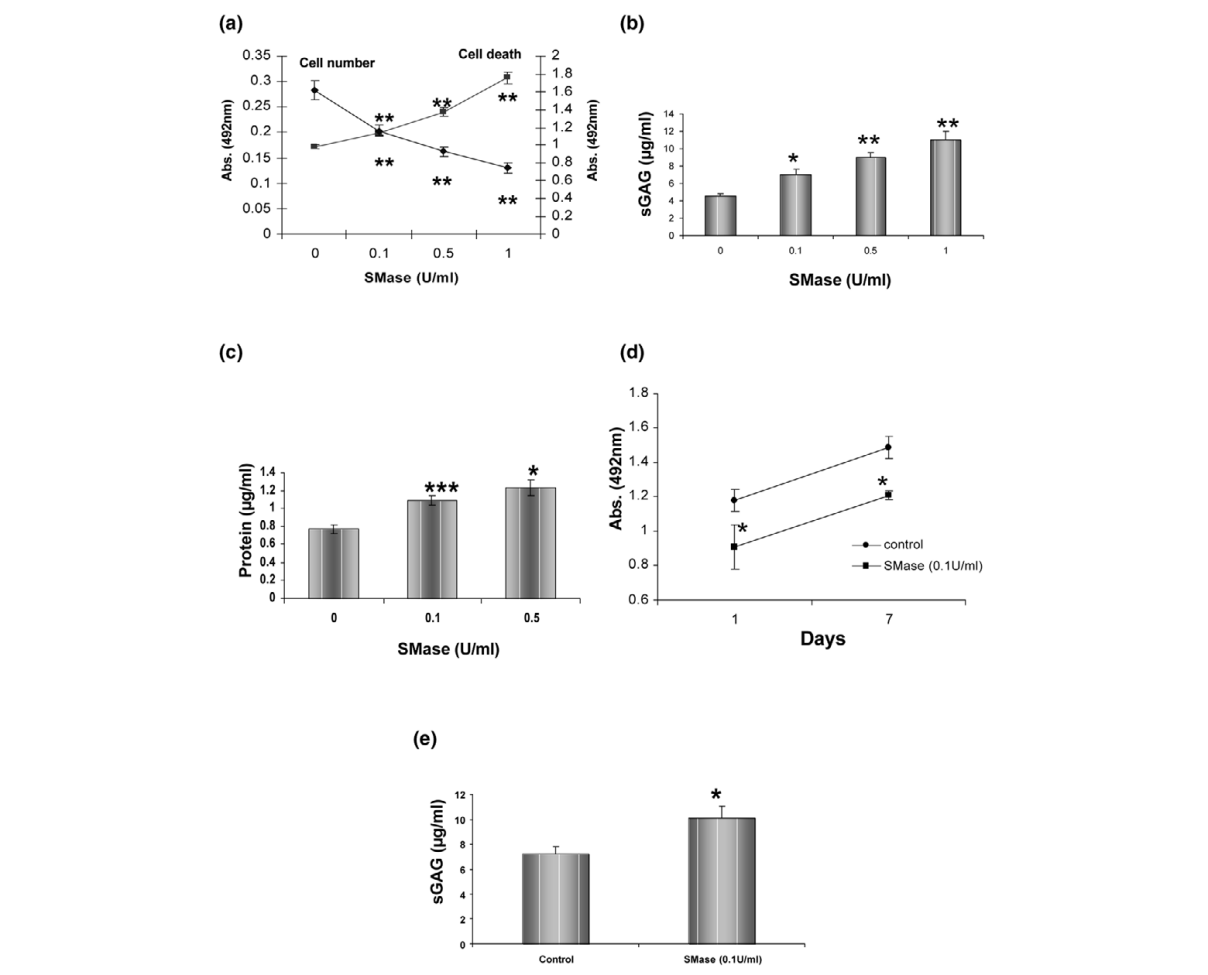
Effect of sphingomyelinase on chondrocyte function. **(a) **Sphingomyelinase treatment dose dependently induces cell death and decreases cell number. Chondrocytes were cultured for 24 hours in the presence of SMase (0–1.0 U/ml). Cell death and cell number were assessed using the CytoTox 96^® ^assay (Promega), which quantitatively measures lactate dehydrogenase released into the media upon cell death during the culture period or upon lysis of living cells at the end of the culture period. Data shown are mean absorbance units (492 nm) ± standard error. ** *P *< 0.01 versus control. **(b) **Short-term SMase treatment induces proteoglycan release from articular chondrocytes. Chondrocytes were cultured for 24 hours in the presence of SMase (0–1.0 U/ml). Media was analyzed for release of sGAGs using the DMMB assay. Differences in release of sGAG associated with culture treatment are expressed as mean sGAG released per cell (mg/ml) ± standard error. * *P *< 0.05; ** *P *< 0.01. **(c) **SMase dose dependently increases cellular protein content. Following 24 hours of treatment with increasing doses of SMase, cells (with cell-associated matrix proteins) were solubilized with 0.9% Triton X-100 and the protein content (mg/ml) determined using the BCA assay (Pierce). Data are presented as mean ± standard error. * *P *< 0.05; *** *P *< 0.001, versus control. **(d) **Long-term SMase treatment reduces cell proliferation. Chondrocytes were cultured for 1–7 days and the effect of SMase (0.1 U/ml) on cell number determined using the CytoTox^® ^assay. Data shown are mean absorbance units (492 nm) ± standard error. * *P *< 0.05 versus control at equivalent time point. **(e) **Long-term SMase treatment induces proteoglycan release from articular chondrocytes. Chondrocytes were cultured in the presence of SMase (0.1 U/ml) for 7 days. The amount of sGAG released into the media per cell was determined as above. Data are presented as mean ± standard error. * *P *< 0.05. DMMB, dimethylmethylene blue; sGAG = sulphated glycosaminoglycan; SMase, sphingomyelinase.

Because there are no previous studies investigating the effect of exogenous SMase on chondrocyte function, we first determined its effect at different concentrations. Chondrocytes were cultured with a range of SMase concentrations (0–1.0 U/ml) for 24 hours and the cells assessed for viability, sGAG and protein release (Figure 2). SMase caused a dose-dependent increase in chondrocyte death with a concomitant decrease in cell number (Figure 2a). The amount of sGAG released into the media following 24 hours of treatment was measured using the DMMB assay (Figure 2b). SMase treatment caused a significant, dose-dependent increase in sGAG release into the media. Cell extracts with associated matrix from SMase-treated cultures contained significantly more protein than did untreated controls (0.1 U/ml, *P *< 0.001; 0.5 U/ml, *P *= 0.024; Figure 2c).

A dose of 0.1 U/ml was chosen for further study because this caused a minimal level of cell death at 24 hours (control 0.17 ± 0.0003 versus SMase 0.2 ± 0.005). An identical experiment was thus performed and cells cultured for 1–7 days. Cell number, cell death and the amount of sGAG released into the media over this period were measured. Over 7 days, an equivalent level of cell death was observed in all cultures regardless of treatment and did not exceed 10–15% of the total cell number (data not shown). Despite this, over the same culture period, significantly fewer cells were found in SMase-treated cultures than in controls (*P *= 0.049), suggesting reduced proliferation (Figure 2d). In addition, SMase significantly increased the amount of sGAG released in to the media (*P *= 0.046; Figure 2e).

### Effect of SMase on *de novo *sGAG and collagen synthesis

**Figure 3:**
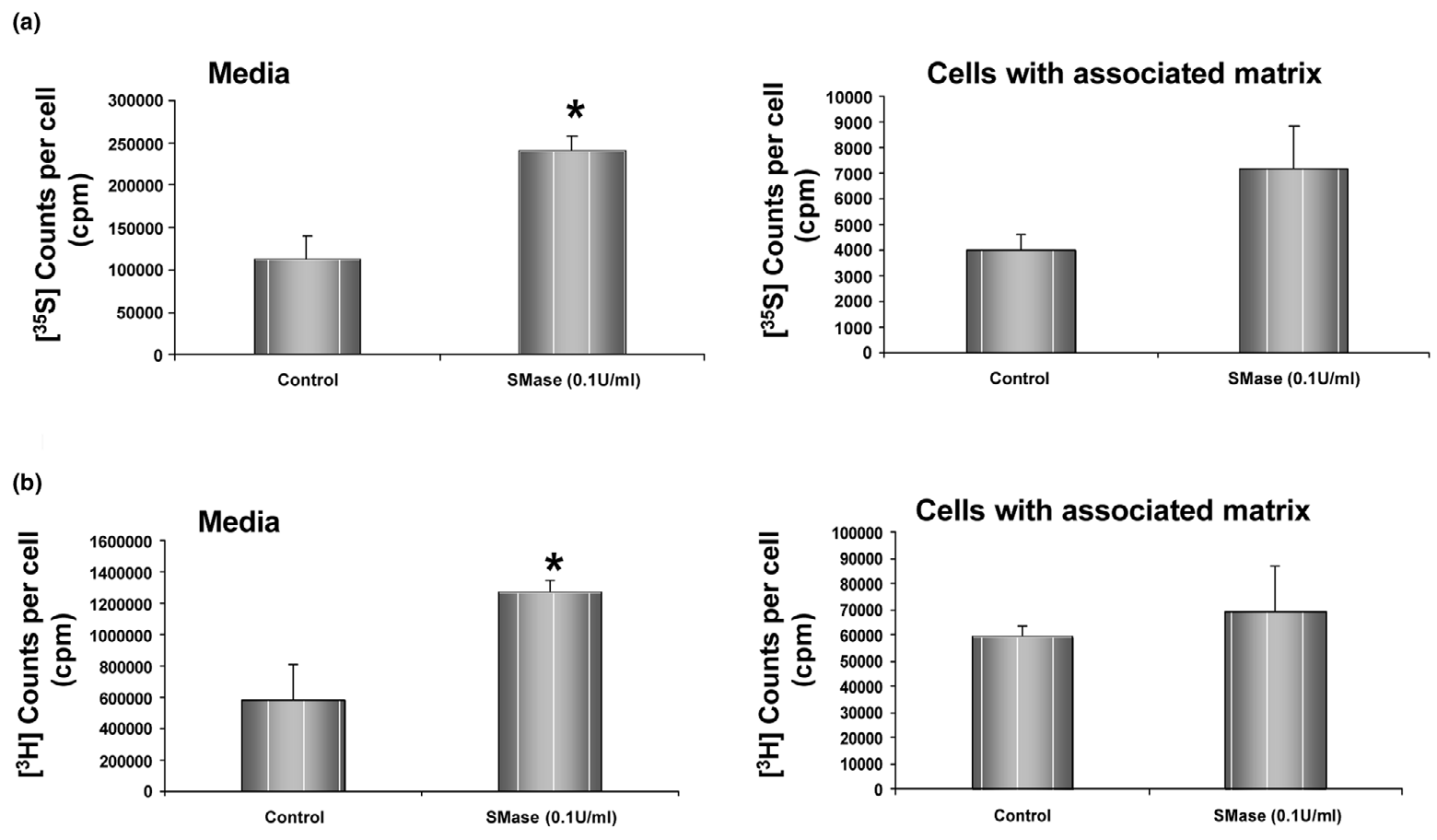
Sphingomyelinase increases *de novo *sGAG and collagen synthesis in articular chondrocytes. Bovine articular chondrocytes were cultured for 7 days with 20 μCi/ml [^3^H]-proline and 10 μCi/ml [^35^S]-sulphate in the presence or absence of SMase (0.1 U/ml). At the end of the culture period, unincorporated label was removed and **(a) **[^35^S] counts (cpm) were measured in cell associated material and media as a measure of *de novo *sGAG. *De novo *collagen synthesis was determined by digesting labelled protein in media and cell extracts with 8U bacterial collagenase overnight at 37°C. Digested collagen fragments were removed using Ultrafree^®^-MC filter units and remaining [^3^H] counts taken as a measure of noncollagenous protein. **(b) **Collagenous protein was calculated using the following equation: collagen (cpm) = total protein ([^3^H] counts before collagenase digestion) – noncollagenous protein (counts remaining after collagenase digestion). Data are normalized to cell number and presented as mean ± standard error. * *P *< 0.05 versus control. cpm, counts/min; sGAG, sulphated glycosaminoglycan.

To determine whether the observed SMase-mediated increase in sGAG and protein release into the media was due to increased synthesis, radiolabelling experiments were performed. Cultures were treated with SMase (0.1 U/ml) for 7 days in the presence of 10 μCi/ml [^35^S]-sulphate and 20 μCi/ml [^3^H]-proline. In addition to measurements of total protein, cell extracts and media were digested with collagenase to determine what proportion of the *de novo *protein synthesised was collagen. At the end of the culture period, unincorporated label was removed and [^35^S] counts (counts/min) measured in cell associated material and media as a measure of *de novo *sGAG (Figure 3a). SMase did not significantly increase the amount of newly synthesized sGAG associated with the cell but did significantly increase the level of newly synthesized sGAG in the media (*P *= 0.017). SMase significantly enhanced the amount of *de novo *collagen released into the media (*P *= 0.015; Figure 3b).

### Investigation of chondrocyte phenotype following culture with SMase

**Figure 4:**
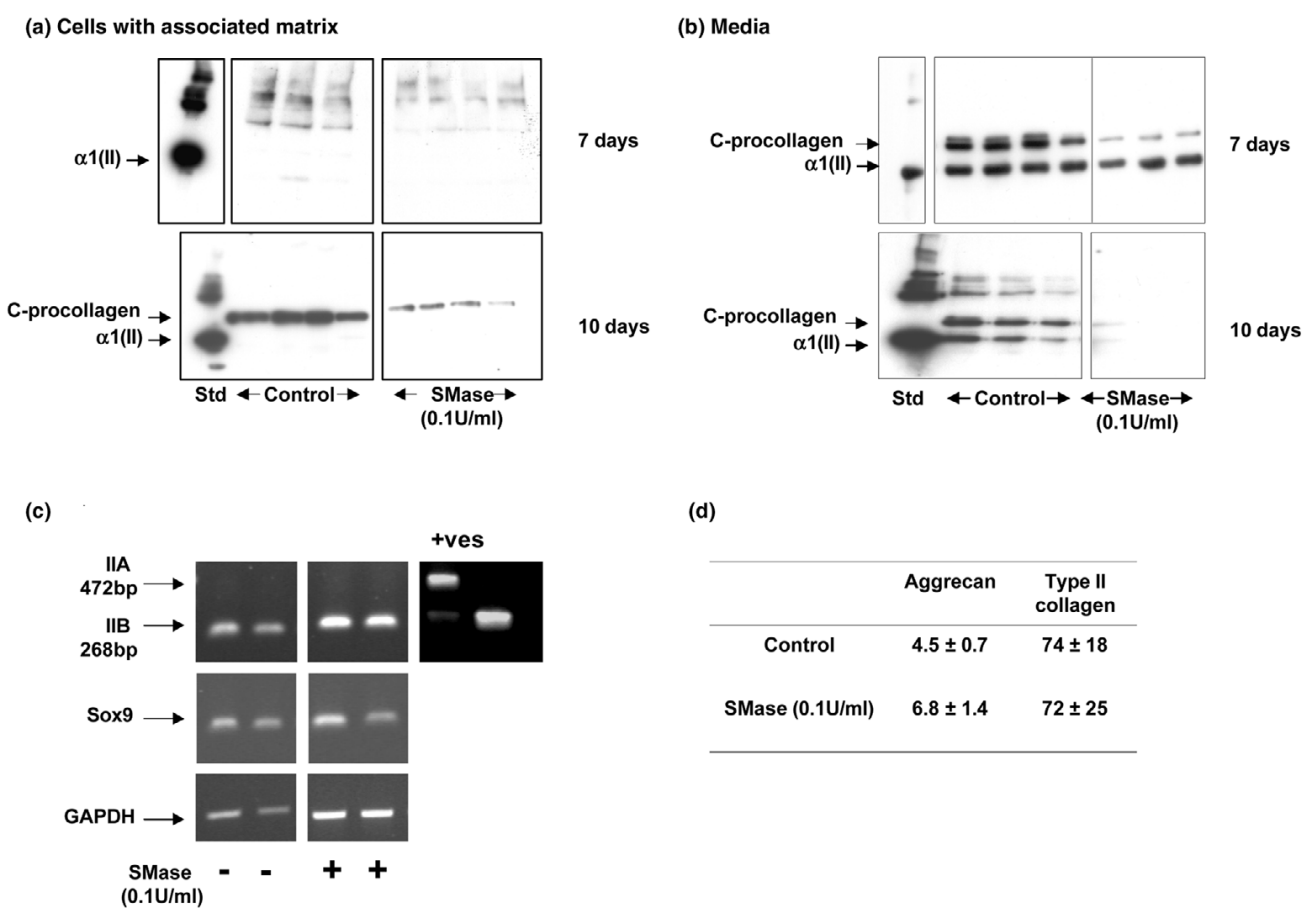
A differentiated chondrocyte phenotype is maintained but sphingomyelinase treatment reduces type II collagen expression. Bovine articular chondrocytes were cultured as monolayers for 7–10 days in ITS supplemented media in the presence or absence of SMase (0.1 U/ml). Where cultures were extended to 10 days, media and treatments were refreshed at day 7. **(a) **Equivalent numbers of cells and their associated matrix and **(b) **media were resolved on 7.5% (weight/vol) SDS-PAGE gels. Samples were analyzed for type II collagen by Western blotting using our monoclonal antibody (AVT6E3). In addition, cells cultured in the presence (+) or absence (-) of SMase (0.1 U/ml) for this period were placed into TRIZOL^® ^(1 × 10^6 ^cells/ml) and total RNA extracted, in accordance with the manufacturer's instructions. **(c) **cDNA was generated and PCR performed using primers to type IIA and IIB procollagen and Sox9. cDNA integrity was confirmed using primers to GAPDH. **(d) **The relative expression level, normalized to GAPDH, of aggrecan and type II collagen mRNAs was determined by quantitative PCR. Data are presented as mean ± standard error. α1(II), α1 chain type II collagen. bp, base pairs; GAPDH, glyceraldehyde-3-phosphate dehydrogenase; ITS, insulin-transferrin-sodium selenite; RT-PCR, reverse transcription polymerase chain reaction; SMse, sphingomyelinase.

Type II collagen is the major collagen component of articular cartilage and is considered a marker for the differentiated chondrocyte phenotype. Two forms are generated by alternative mRNA splicing, namely types IIA and IIB, which include and exclude exon 2, respectively. The shift from IIA to IIB accompanies chondrocyte differentiation, whereas re-expression of IIA procollagen has been reported in osteoarthritic cartilage, indicating the potential reversion of the cells to a chondroprogenitor cellular phenotype [34]. There was no apparent difference in cell morphology with any of the treatments. Following 7–10 days of culture, cell extracts with their associated matrix and media were analyzed for type II collagen by Western blotting (Figure 4). Control cells produced type II procollagen, processing it (α1 [II]) and secreting it into the media (Figure 4a,b), demonstrating that the differentiated chondrocyte phenotype was maintained in our culture system. In contrast, SMase treatment decreased the amount of type II procollagen (Figure 4a,b) as well as resulting in a reduction in the level of processed collagen in the media (Figure 4b). This response was further enhanced at day 10 following an application of fresh SMase at day 7. RT-PCR analysis of chondrocytes after 7 days showed that the mRNAs for the phenotypic markers of articular cartilage chondrocytes, type IIB collagen and Sox9 were expressed in both control and SMase treated cells (Figure 4c). SMase treatment had no significant effect on type II collagen or aggrecan mRNA expression normalized to GAPDH (Figure 4d).

### Effect of inhibiting activation of PKR on cartilage matrix homeostasis

**Figure 5:**
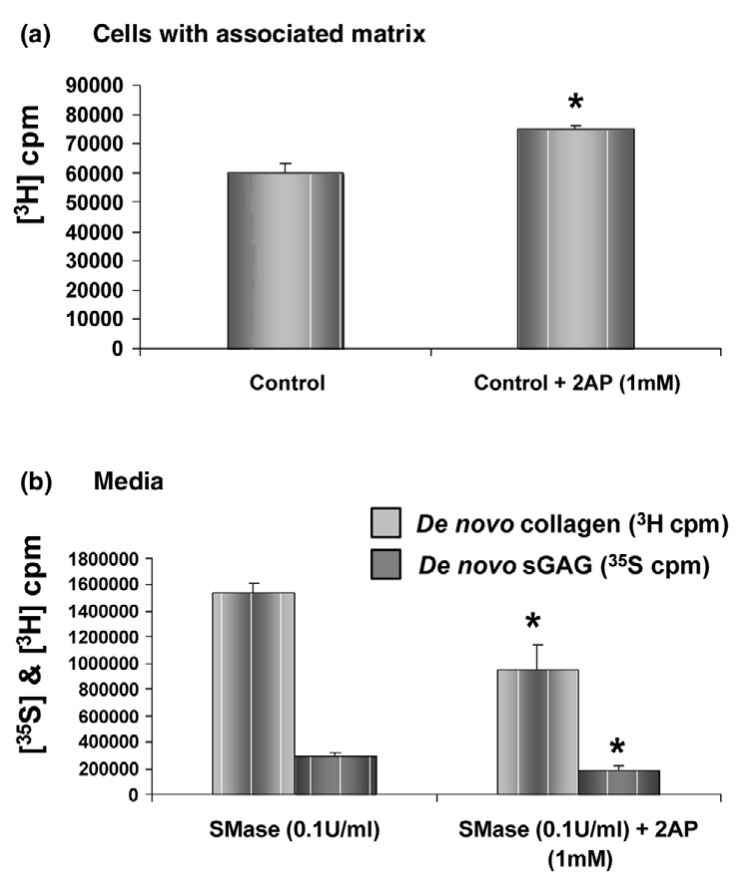
PKR is involved in cartilage matrix homeostasis. To determine whether PKR mediates the observed changes in chondrocyte matrix homeostasis, PKR activity was inhibited by adding 1 mmol/l 2AP to duplicate cultures. **(a) **Inhibition of PKR in untreated, control cells caused an increase in cell associated collagen. **(b) **Addition of 2AP to sphingomyelinase-treated cultures resulted in a significant reduction in the amount of *de novo *sGAG and collagen in the media. Data are presented as mean ± strandard error. * *P *< 0.05. 2AP, 2-aminopurine; cpm, counts/min; PKR, protein kinase R; sGAG, sulphated glycosaminoglycan.

The role of PKR in chondrocyte ECM homeostasis was investigated by treating duplicate cultures with the PKR inhibitor 2AP (Figure 5). Inhibition of PKR activity in untreated control cells significantly increased the level of *de novo *collagen associated with the cell (Figure 5a; *P *= 0.018) but had no effect on the amount measured in the media (data not shown). Addition of 2AP in conjunction with SMase did not significantly alter cell death, *de novo *collagen and sGAG associated with the cell (data not shown), but significantly reduced the total amount detected in the media compared with treatment with SMase alone (collagen, *P *= 0.042; sGAG, *P *= 0.042; Figure 5b).

### Both acidic and neutral sphingomyelinases are expressed by articular chondrocytes

**Figure 6:**
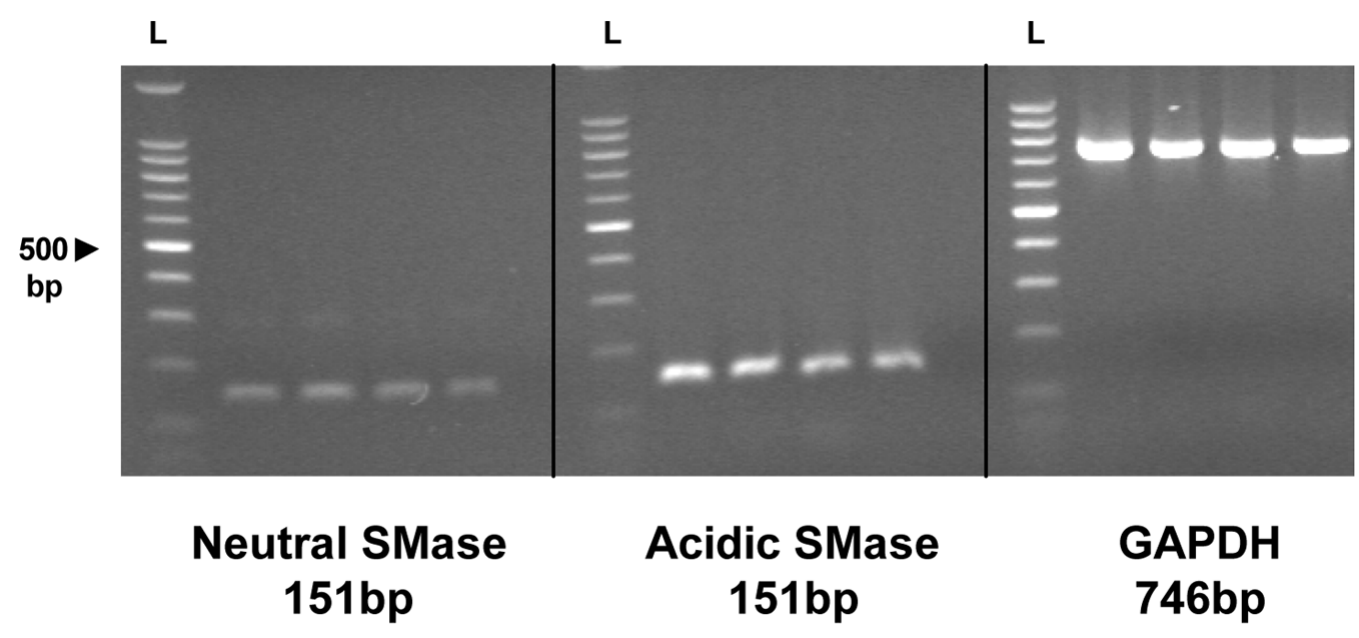
Both neutral and acidic SMase mRNAs are expressed by articular cartilage chondrocytes. Following 24 hours of culture, cells were placed into TRIZOL^® ^(1 × 10^6 ^cells/ml) and total RNA extracted, in accordance with the manufacturer's instructions. cDNA was generated (*n *= 4) and PCR performed using primers specific to GAPDH, or acidic or neutral SMase (Table 1). Amplified products were separated alongside a 100 bp DNA ladder (L) on 1–2% agarose gels, containing ethidium bromide (10 μg/ml). Product sizes (bp) are indicated. bp, base pairs; GAPDH, glyceraldehyde-3-phosphate dehydrogenase; PCR, polymerase chain reaction; SMase, sphingomyelinase.

To investigate whether bovine articular chondrocytes may potentially signal via endogenous SMases, we determined mRNA expression for acidic and neutral sphingomyelinase. RT-PCR revealed that both acidic and neutral SMase mRNAs are expressed by primary articular chondrocytes (Figure 6).

## Discussion

This study demonstrates for the first time that ECM homeostasis in articular cartilage chondrocytes can be profoundly altered by triggering the ceramide signalling pathway. Over 24 hours, raising endogenous levels of ceramide in articular cartilage chondrocytes by treatment with 0.1 U/ml bacterial SMase caused a dose-dependent increase in cell death with a concomitant decrease in cell number. This is in accordance with the known role for ceramide in initiating a cellular stress response resulting in cell death [12]. It should be noted that the assay used to measure cell death in this study detects loss of membrane integrity and thus measures necrosis, either primary or secondary (cultured cells that are undergoing apoptosis *in vitro *eventually undergo secondary necrosis). Therefore, further studies are necessary to determine the extent of apoptotic cell death. Over the extended culture period, SMase treatment resulted in a further reduction in cell number compared with that in control cultures, with the majority of the decrease occurring in the early stages of the treatment; thereafter the rate of proliferation was similar to that in controls (Figure 1d). Because there was no concomitant increase in cell death, this suggests that SMase treatment also decreased chondrocyte proliferation. This in accordance with studies in human keratinocytes, which have shown that a rapid (15 minutes) but transient (returning to baseline by 1 hour) increase in endogenous ceramide occurs following treatment with 0.1 U/ml neutral SMase followed by reduced cellular proliferation over 6 days, the extent of which was equivalent to that seen in the present study [35].

Data obtained from the DMMB assay indicated that SMase increased the release of sGAG from articular chondrocytes. Because this assay does not discriminate between whole sGAG and degraded sGAG fragments, we used incorporation of [^35^S] to determine whether low concentrations (0.1 U/ml) of exogenous SMase affected sGAG synthesis or degradation. As well as increasing sGAG synthesis, SMase also significantly enhanced the level of *de novo *collagen and total protein in the media over seven days of culture, suggesting that SMase acts on chondrocytes to increase expression of ECM components. The hydrolysis of sphingomyelin by the action of SMases is the primary mechanism for rapidly increasing ceramide levels in the cell [36]. As discussed above, at the concentration (0.1 U/ml) used, SMase induces a rapid but transient rise in endogenous ceramide in human keratinocytes [35]. Our data correlate with recent studies in fibroblasts that showed that low doses of ceramide stimulate collagen production [15]. This is contrast to the effect caused by high ceramide, which is thought to inhibit collagen production [15,17,18] because of its conversion to sphingosine-1-phosphate or other inhibitory intermediates, thus promoting anticeramide affects [15].

When chondrocytes are cultured as monolayers on plastic they rapidly de-differentiate, losing expression of type II collagen. More specifically they shift their expression from type IIB to type IIA procollagen [37]. Our monolayer cultures supplemented with ITS retained expression of the normal chondrocyte markers Sox9, aggrecan and type IIB collagen. These were still expressed by SMase-treated chondrocytes with no detectable expression of type IIA mRNA. However, SMase reduced type II collagen protein expression (Western blot), despite increasing total collagen production (^3^[H]-proline incorporation) and maintaining *col2a1 *mRNA expression (qPCR). Therefore, although low levels of endogenous ceramide in chondrocytes appeared to push the homeostatic balance toward ECM synthesis, which is in accordance with studies in fibroblasts [15], this may have been at the expense of type II collagen expression.

Preliminary work within our laboratory suggests that the SMase-induced increase in total collagen production is not due to increases in type I or III collagen, but further investigation is clearly warranted. We propose that small increases in cellular ceramide, as mimicked here, may contribute to the increases in proteoglycan and collagen synthesis [38-40] that are observed in the 'biosynthetic phase' in early osteoarthritis [8]. Given that excessive ceramide accumulation within cartilage is known to produce an osteoarthritis-like phenotype [16], we hypothesize that treatment of chondrocytes with high doses of SMase would result in an accumulation of endogenous ceramide levels within the cells and that it is this that signals downstream to promote cartilage degradative events. This idea that high levels of ceramide promote cartilage degeneration is supported by our earlier studies in which application of C_2_-ceramide increased MMP expression and activation and proteoglycan release from articular cartilage explants [8]. Thus, further investigations to relate levels of ceramide, sphingosine and sphingosine-1-phosphate to chondrocyte ECM synthesis and degradation are clearly needed to determine how the current data fit into the notion of a 'sphingolipid rheostat' [13].

Because our previous studies showed that the protein kinase PKR plays a pivotal role in cartilage homeostasis [8], we inhibited PKR activity to determine whether PKR is involved in the observed changes in matrix synthesis. In control cells, inhibition of PKR caused a significant increase in *de novo *protein synthesis found within the cell and associated matrix but no change in the level released into the media. This is in keeping with the known role played by PKR as an inhibitor of translation [41]. However, inhibition of PKR activity in SMase-treated chondrocytes significantly reduced the amount of newly synthesized sGAG and collagen detected in the media, suggesting a role for PKR in SMase-induced matrix synthesis. Because high levels of ceramide have previously been shown to result in PKR-mediated inhibition of protein synthesis in a leukaemia cell line [4], this would suggest that a complex interplay of signalling pathways are involved in SMase-mediated PKR signalling in chondrocytes, the exact nature of which remains to be elucidated.

Finally, we showed, for the first time, that articular chondrocytes can express both acidic and neutral SMases and so are able, given the appropriate external signal, to raise levels of endogenous ceramide. It has been shown that TNF-α can increase cellular ceramide levels via the *de novo *pathway as well as by binding to its membrane receptor (TNFR55), causing activation of neutral or acidic SMase [36,42,43]. Depending on which SMase is activated, an inflammatory (neutral SMase) or apoptotic (acidic SMase) response then occurs. Because TNF-α levels are elevated in arthritis and TNFR55 expression is increased in arthritic disease [44], our future studies will determine whether TNF-α-mediated activation of neutral SMase and ceramide generation plays a role in cartilage degradation.

## Conclusion

In the present study we found that sphingomyelinase, at low concentration, stimulated ECM synthesis in articular chondrocytes, and this was in part mediated by PKR. Importantly, the increase in collagen production was not due to increases in type II collagen. Therefore, small increases in endogenous ceramide in chondrocytes appear to push the homeostatic balance toward ECM synthesis but at the expense of the chondrocytic phenotype. We therefore hypothesize that during the 'biosynthetic phase' in early osteoarthritis, the observed increases in proteoglycan and collagen synthesis may be due to a small increase in cellular ceramide triggered by circulating cytokines such as TNF-α via activation of PKR. Excessive ceramide accumulation may then play a role in the later stages of cartilage degradation.

## Abbreviations

2AP = 2-aminopurine; DMEM = Dulbecco's modified eagle's medium; DMMB = dimethylmethylene blue; ECM = extracellular matrix; GAPDH = glyceraldehyde-3-phosphate dehydrogenase; ITS = insulin-transferrin-sodium selenite; LDH = lactate dehydrogenase; MMP = matrix metalloproteinase; PKR = protein kinase R; RT-PCR = reverse transcription polymerase chain reaction; sGAG = sulphated glycosaminoglycan; SMase = sphingomyelinase; TNF = tumour necrosis factor.

## Competing interests

The authors declare that they have no competing interests.

## Authors' contributions

SJG conceived the study, generated most of the data and drafted the manuscript. EJB helped in the conception of the study, generated the QPCR data and made substantial contributions to the acquisition of the radiolabelling data. PJ was involved in the acquisition of some of the toxicity and sGAG data. EJB, VCD and DJM helped in the interpretation of data and were involved in revising the manuscript. All authors read and approved the final manuscript.

## Figures and Tables

**Table 1:** PCR primers

Gene	Strand	Sequence	Annealing temp (°C)	MgCl_2 _(mmol/l)	Product size (bp)	Reference/GenBank accession number
Acidic SMase	Forward	5'-ATCGGCCTTAATCCTGGTGA-3'	58	2.5	151	AF325550
	Reverse	5'-GACTGGACACGGAGAGGGC-3'				
Neutral SMase	Forward	5'-TTGGCAGTGGCCTCTGTGTG-3'	58	2.5	151	NM031360
	Reverse	5'-AGTCCGCTTAGATGGAGCACC-3'				
GAPDH	Forward	5'-TGGTCACCAGGGCTGCTTTTA-3'	60	2.5	746	U85042
	Reverse	5'-CGCCTGCTTCACCACCTTCT-3'				
Sox9	Forward	5'-GGGCGAGCCGGACCTGAAGAA-3'	53	1.5	321	AF278703
	Reverse	5'-CGCTCCGCCTCCTCCACGAAC-3'				
Col2a1	Forward	5'-GCCTCGCGGTGAGCCATGATC-3'	60	1.5	IIA: 472	Valcourt and coworkers [30]
	Reverse	5'-CTCCATCTCTGCCACGGGGT-3'			IIB: 268	

*Unless previously published, primers were designed to GenBank sequences using Primer Express^® ^software (Applied Biosystems). bp, base pairs; SMase, sphingomyelinase.
